# *Vat*, an Amazing Gene Conferring Resistance to Aphids and Viruses They Carry: From Molecular Structure to Field Effects

**DOI:** 10.3389/fpls.2016.01420

**Published:** 2016-09-26

**Authors:** Nathalie Boissot, Alexandra Schoeny, Flavie Vanlerberghe-Masutti

**Affiliations:** ^1^Génétique et Amélioration des Fruits et Légumes, INRAMontfavet, France; ^2^Pathologie Végétale, INRAMontfavet, France; ^3^Centre de Biologie pour la Gestion des Populations, UMR CBGP, INRAMontferrier-sur-Lez, France

**Keywords:** NLR resistance gene, durability, melon, *Cucumis melo*, Aphis gossypii, resistance deployment, resistance to insects, resistance to viruses

## Abstract

We review half a century of research on *Cucumis melo* resistance to *Aphis gossypii* from molecular to field levels. The *Vat* gene is unique in conferring resistance to both *A. gossypii* and the viruses it transmits. This double phenotype is aphid clone-dependent and has been observed in 25 melon accessions, mostly from Asia. It is controlled by a cluster of genes including CC-NLR, which has been characterized in detail. Copy-number polymorphisms (for the whole gene and for a domain that stands out in the LLR region) and single-nucleotide polymorphisms have been identified in the *Vat* cluster. The role of these polymorphisms in plant/aphid interactions remains unclear. The *Vat* gene structure suggests a functioning with separate recognition and response phases. During the recognition phase, the VAT protein is thought to interact (likely indirectly) with an aphid effector introduced during cell puncture by the aphid. A few hours later, several miRNAs are upregulated in *Vat* plants. Peroxidase activity increases, and callose and lignin are deposited in the walls of the cells adjacent to the stylet path, disturbing aphid behavior. In aphids feeding on *Vat* plants, Piwi-interacting RNA-like sequences are abundant and the levels of other miRNAs are modified. At the plant level, resistance to aphids is quantitative (aphids escape the plant and display low rates of reproduction). Resistance to viruses is qualitative and local. Durability of NLR genes is highly variable. *A. gossypii* clones are adapted to *Vat* resistance, either by introducing a new effector that interferes with the deployment of plant defenses, or by adapting to the defenses it triggered. Viruses transmitted in a non-persistent manner cannot adapt to *Vat* resistance. At population level, *Vat* reduces aphid density and genetic diversity. The durability of *Vat* resistance to *A. gossypii* populations depends strongly on the agro-ecosystem, including, in particular, the presence of other cucurbit crops serving as alternative hosts for adapted clones in fall and winter. At the crop level, *Vat* resistance decreases the intensity of virus epidemics when *A. gossypii* is the main aphid vector in the crop environment.

## Introduction

Host-plant resistance is an effective, environmentally friendly means of controlling insect pests, including aphids. Here, we consider plant resistance to be a heritable trait, displaying genotype-dependent variability within a plant species. Resistance to aphids has been described in several crops (Dogimont et al., 2010; Smith and Chuang, 2014). This resistance is controlled by one or several genes, which may be recessive or dominant. Resistance deters aphids from the crop, and affects their biotic potential, including their growth, development, and reproduction. So resistance is generally detected through these central aphid life history traits, rather than by a visible plant phenotype. The melon *Vat* gene is unique among the known resistance genes in that it has a pleiotropic effect as it also confers resistance to the viruses transmitted by aphids.

Melon crops are primarily colonized by only one aphid species, the melon aphid *Aphis gossypii*, a cosmopolitan aphid species. This aphid causes stunting and severe leaf-curling, and heavy colonization can result in plant death. Aphids also excrete honeydew onto the leaves and fruits. This sticky sweet substance acts as an ideal growth medium for sooty mold, which greatly decreases fruit quality. Moreover, *A. gossypii* is an efficient vector for viruses, contributing to the spread of diseases.

Resistance to *A. gossypii* in melon was first observed in the mid-20th century (Ivanoff, 1944). In 1967, an American team of entomologists and plant geneticists began a systematic study of resistance to *A. gossypii* in melon. They focused on the Indian line PI 371795, later called PI 414723, which suffers only mild attacks in the field (Kishaba et al., 1971; Bohn et al., 1972). In controlled no-choice tests, few aphids survive on this line, and the fecundity of those that do is low (Kishaba et al., 1971). This resistance is a dominant trait in PI 414723, and is controlled by a major gene and several minor genes (Kishaba et al., 1976). A French team of virologists and plant geneticists studied the resistance of the Korean line PI 161375 to *Cucumber mosaic virus* (CMV) in detail. They discovered an original phenotype of this line: complete resistance to CMV when the aphid *A. gossypii* inoculated the plant with the virus. Moreover, *A. gossypii* aphids departed from PI 161375 plants. These two phenotypes cosegregated in PI 161375 and were controlled by a single dominant gene (Pitrat and Lecoq, 1980). Complete resistance to CMV was also observed in PI 414723 when CMV was introduced into the plant by the aphid *A. gossypii* (Pitrat and Lecoq, 1982). PI 414723 and PI 161375 thus have similar features: resistance to CMV when *A. gossypii* inoculates the plant with the virus cosegregating with resistance to *A. gossypii* controlled by a single dominant gene (Pitrat and Lecoq, 1982). In both lines, the resistance to viruses is expressed only if the aphid inoculating the plant with the virus is *A. gossypii*. PI 161375 and PI 414723 plants are susceptible to viruses when other aphid species, such as *A. citricola, A. craccivora, A. fabae*, and *Myzus persicae*, inoculate the plant with viruses, or if viruses are introduced mechanically (Lecoq et al., 1979, 1980; Romanow et al., 1986). The resistance to viruses when *A. gossypii* inoculated the plant is also fully effective against unrelated viruses (Lecoq et al., 1980). The gene controlling this double phenotype has been named *Vat*, for ‘virus aphid transmission’ (Pitrat and Lecoq, 1982).

Several hundreds of accessions were tested for their effect on the aphid traits (Pitrat et al., 1996; Fergany et al., 2011). These large screenings have suggested that about 5% of accessions display resistance to colonization by *A. gossypii*. Among them, only a small number have been tested for the double phenotype characteristic of *Vat*, resistance to virus and resistance to aphids. Up to now, the double phenotype has been identified in 25 melon lines (Pitrat and Lecoq, 1980; Soria et al., 2000; Thomas et al., 2012b; Boissot et al., 2016). These melon accessions or lines originate from Asia, Africa, America, and Europe.

Two independent breeding programs were conducted early on, to transfer resistance to *A. gossypii* into cultivars, with the transfer of resistance from PI 161375 into Charentais-type melons and resistance from PI 414723 into Western Shipper–type melons. Consistent with the cosegregation of resistance to melon aphid and resistance to viruses, which were introduced by melon aphids, the inbred lines obtained in both programs also displayed resistance to viruses when the melon aphid inoculated the plant (Kishaba et al., 1992; Boissot et al., 2016). Margot became the first melon cultivar declared resistant to the melon aphid *A. gossypii* to be listed in the French catalog in 1987. Since then, 110 Charentais-type cultivars declared resistant to this aphid have been released in France (GEVES data). Melons are cultivated in the South East (SE) and South West (SW) of France, and on two islands of the Lesser Antilles (LA). Given the commercial success of some of the resistant cultivars, about 80% of the melon crops cultivated in SE France since 2000 are thought to have carried this resistance (Boissot et al., 2016).

Since these seminal studies were conducted, the molecular structure of the *Vat* gene has been elucidated, its double phenotype has been investigated at the cellular level, and its effect on the behavior and life-history traits of the aphid has been studied. Its spectrum of activity against the clonal diversity of *A. gossypii* has been studied in the laboratory, and its efficacy and the durability of these effects have been studied *in situ*. All these points will be reviewed after a short presentation of the three protagonists*: Cucumis melo, A. gossypii* and the viruses transmitted by *A. gossypii*.

## *Cucumis melo, Aphis gossypii* and the Viruses it Transmits to Melon

### *Cucumis melo* Shares a Number of Features Specific to Cucurbits, but is Genetically Isolated in its Family

*Cucumis melo* is one of the principal species from the Cucurbitaceae family. Asia is its geographic region of origin and it belongs to the *C. melo*/*C. callosus*-*C. trigonus* complex, which diverged 3 million years ago (Mya) from an Australian sister species, *C. picrocarpus* (Sebastian et al., 2010). This clade diverged from the lineage leading to cucumber (*C. sativus*) about 10 Mya. A highly effective reproductive barrier now isolates *C. melo* from most of its relatives, with successful crosses reported only with *C. hystrix* (Chen and Adelberg, 2000). Based on data for polymorphism at simple sequence repeat (SSR) markers, *C. melo* split into two main genetic clusters (**Table 1**), the first containing four groups (A, B, C, D) and the second containing three groups (E, F, and G; Serres-Giardi and Dogimont, 2012). These data, together with findings for chloroplast polymorphisms (Tanaka et al., 2013), suggest that there were two or three domestication events, one in Asia, another in Africa or Western Asia, and a third in Africa (Pitrat, 2013).

**Table 1:** List of melon lines exhibiting the double phenotype, resistance to aphids and resistance to viruses when the aphids inoculate the plant, and multilocus genotypes (MLGs) of *Aphis gossypii* clones revealing that phenotype.

Characteristic of melon Accessions						Characteristics of aphid clones^c^		
Genetic groups^a^	Botanical groups^b^	Asia	Africa	America	Europe	I	II	III
(I) A	Inodorus				Anso 77		CUCU3	NM1
(I) A	Inodorus				Invernizo 8427			NM1
(I) A	Reticulatus		PI 224770					NM1
(I) B	Flexuosus		Fegouss 1					NM1
(I) B	unknown	San Ildefonso					CUCU3	NM1
(I) C	unknown	Durgapura Madhu				C9		NM1
(I) D	Makuwa	Kanro Makuwa 1				C9		NM1
(I) D	Makuwa	Kanro Makuwa 2				C9		NM1
Unknown	Momordica	AM 51				C9, CUC1, GWD	CUCU3	NM1
(II) E	Momordica	PI 414723				C9		NM1
(II) E	Wild		PI 482398			C9, GWD	CUCU3	NM1
(II) E	Wild		HSD2455				CUCU3	
(II) F	Acidulus		PI 482420			C9		NM1
(II) F	Acidulus	90625						NM1
(II) F	Acidulus	PI 164723						NM1
(II) F	Chito			Meloncillo				NM1
(II) G	Chinensis	Chenggam						NM1
(II) G	Chinensis	Miel Blanc						NM1
(II) G	Chinensis	PI 161375					CUCU3	NM1
(II) G	Chinensis	PI 255478				C9		NM1
(II) G	Chinensis	PI 266935						NM1
(II) G	Conomon	Shiro Uri Okayama						NM1
(II) G	Makuwa	K 5442				C9		NM1
(II) G	Makuwa	Ginsen Makuwa				C9		NM1
(II) G	Makuwa	Shirokawa Nashi Makuwa						NM1

*^a^According to Serres-Giardi and Dogimont (2012). ^b^According to Pitrat et al. (2000). ^c^According to Thomas et al. (2016).*

*Cucumis melo* is now found throughout the world and, like many crops, cultivated melons display extensive phenotypic polymorphism, defining botanical groups, whereas wild melons display low levels of phenotypic polymorphism (Pitrat et al., 2000). The first evidence of *C. melo* domestication date to just after 3000 BC, in China and Egypt (Pitrat, 2003). Diversification after domestication is controlled mostly by recessive traits, such as sex expression, fruit shape, vein tracts, number of placentas, a gelatinous sheath around the seeds, and white flesh color, whereas disease resistance is mostly conferred by dominant genes (Pitrat, 2013). Melon is now an important fruit crop, with 16 commercial melon types identified by the Organization for Economic Co-operation and Development (OECD) guidelines on the basis of fruit characteristics (shape, skin color and surface characteristics, color of the flesh and dehiscence of the peduncle). Twenty-five to 30 million tons of melon have been produced annually over the last 10 years, with about half of this total in China (FAOSTAT database^1^). Melon has been subject to intense selection, and its isolation in the genus *Cucumis* has led to reclaim the broad diversity present in both cultivated and wild forms (Pitrat, 2013). Twenty to 30 new melon cultivars have been added to French catalogs annually since 2000 (GEVES data^2^).

Melon is a diploid species with a relatively small genome (450 Mb) that has recently been fully sequenced (Garcia-Mas et al., 2012). It has 12 chromosomes, and, like all cucurbits, its genome displays no evidence of recent duplication since the eudicot paleotriplication event. It has a small number of resistance genes, only 81 putative NLR genes were identified (Garcia-Mas et al., 2012), possibly reflecting an unusual adaptive strategy in cucurbits potentially involving specific mechanisms of disease resistance gene regulation or the characteristic vascular structure of these plants. Cucurbits have an unusual vascular structure, with two types of phloem: the fascicular phloem is located in the main vascular bundles, and the extra-fascicular phloem is peripheral to the fascicular phloem, dispersed throughout the cortical tissue of the stems and petioles (Zhang et al., 2010). The fascicular phloem is mostly involved in sugar transport, whereas the extra-fascicular phloem may be involved in signaling and the transport of other metabolites.

### *A. gossypii* Glover: A Biotype Specializing on Cucurbits

The *A. gossypii* group diverged from other aphids 12 to 25 Mya, during the radiation period of its host plants (Hyojoong et al., 2011). Within this group, species diversification may have been a rapid and recent process, as suggested by phylogenetic trees based on morphological characters (Kim et al., 2010) and the inability of differentiating between species on the basis of mitochondrial DNA COI/COII (Coeur d’acier et al., 2007). The mitochondrial *Cytb* and *nuclear sodium channel para-type* (*SCP*) genes can be used to distinguish the species *A. gossypii* Glover from related species native to North America, Europe, and Asia (Carletto et al., 2009a; Hyojoong et al., 2011; Lagos-Kutz et al., 2014). Hereafter, we will use the term *A. gossypii* to refer to *A. gossypii* Glover.

*Aphis gossypii* is a cosmopolitan species that is extremely polyphagous, colonizing hundreds of plant species (Ebert and Cartwright, 1997). In northern areas, at latitudes above 30°N, *A. gossypii* produces sexual morphs in the fall, which produce eggs that diapause on its primary hosts (Kring, 1959; Takada, 1988; Ferrari and Nicoli, 1994). These primary hosts differ between geographic areas, with Rose of Sharon (*Hibiscus syriacus*) frequently identified as a host plant in Asia, Europe, and America. In spring and summer, *A. gossypii* becomes a pest on crops, its secondary hosts, on which it reproduces clonally. In intertropical areas, *A. gossypii* reproduces clonally all year round. Thus, depending on the area of melon production, *A. gossypii* populations may consist of a mixture of strictly clonal lineages and lineages derived from sexual reproduction or of strictly clonal lineages only.

*Aphis gossypii*, currently named the cotton-melon aphid, is a pest for several crops, including melon, marrow, zucchini, potato, eggplant, cotton, ornamental hibiscus, and citrus fruit trees. Like all aphids, *A. gossypii* carries the bacterium *Buchnera aphidicola* as an obligate endosymbiont providing several essential nutrients (Douglas, 2003) and the phenotypic plasticity in host plant use by *A. gossypii* may be related to the size of the *B. aphidicola* population (Zhang et al., 2016). Many other facultative endosymbionts have been detected in aphid species and shown to play a role in species ecology (Oliver et al., 2010); however, facultative endosymbionts appear to be rare in *A. gossypii* (Carletto et al., 2008).

A small number of *Cytb* sequence polymorphisms differentiate three haplotypes of *A. gossypii* collected on crops and plants from the Cucurbitaceae, Malvaceae, Solanaceae, and Rosaceae in Africa, South America, Australia, and Europe (Carletto et al., 2009a). All individuals collected from cucurbits belong to the same haplotype. A small number of mitochondrial DNA sequence polymorphisms between the *Cytb* and 16S genes distinguish two biotypes of *A. gossypii* specializing on cotton and cucurbits in North China (Wang et al., 2016). At the end of the 1990s, a set of SSR markers (SSRs) was developed to assess *A. gossypii* diversity (Vanlerberghe-Masutti et al., 1999). Several hundred multilocus genotypes (MLGs), defined on the basis of allelic combinations at eight SSR markers, have since been described. The largest set of MLGs was identified in a study of spring migrants in France and the Lesser Antilles; they formed seven genetic clusters (Thomas et al., 2012a, 2016). All individuals collected from colonies on melon shared MLGs distributed between three clusters (later named in the manuscript I, II, and III). In data analyzed with the same set of reference clones (Brévault et al., 2008; Charaabi et al., 2008; Carletto et al., 2009b; Chen et al., 2013; Thomas et al., 2016), 75 MLGs were observed in colonies collected from cucurbits in Asia, Africa, Europe, Australia, and Caribbean islands, four of which — C4, C11, C9 and NM1 — were observed in at least two geographic areas suggesting that these clusters contain individuals specializing on cucurbits.

Biological studies have been conducted at the laboratory and field levels to assess the strength of the host specialization of *A. gossypii* biotypes. The results of these studies can be related with genetic knowledge. Many laboratory host-transfer experiments have been conducted with plants from the Cucurbitaceae (cucumber), Malvaceae, (cotton, okra, and hibiscus), Solanaceae (eggplant and sweet pepper), and Rutaceae (citrus plants and Chinese prickly ash) (Guldemond et al., 1994; Liu et al., 2008; Carletto et al., 2009b; Satar et al., 2013; Wu et al., 2013; Xu et al., 2014; Wang et al., 2016). Overall, the results obtained suggest that on one hand cucurbit biotypes poorly colonize plants from other plant families, if not at all, with the exception of *H. syriacus* and on the other hand, most, if not all, biotypes specialized on other crops poorly colonize cucurbits. Host switching in the field has been inferred from molecular markers. Studies conducted in different agricultural environments in Africa and China in which cucurbits are present together with cotton, and citrus or Solanaceous crops have confirmed that lineages specializing on cucurbits cannot easily switch to other crops (Brévault et al., 2008; Charaabi et al., 2008; Wang et al., 2016).

Taking into account genetic, ecological and lab experiment data, specializing on cucurbits of a part of *A. gossypii* species is fairly clear. All clones able to colonize cucurbits form an ecological group called the Cucurbit host-race (Carletto et al., 2009b), they are assigned to the genetic clusters I, II, and III (Thomas et al., 2016). The history of co-evolution between *A. gossypii* and cucurbits merits further investigation, particularly as the role of cucurbit’s distinctive phloem structure for *A. gossypii* specialization on these plants.

### The Viruses Transmitted to Melon by *A. gossypii* Belong to Three Families

More than 70 virus species have been reported to attack cucurbits (Lecoq and Katis, 2014). Some cause severe epidemics in melon crops worldwide. Five of these species are transmitted by aphids, including the melon aphid. These viruses may or may not persist in the vector. Non-persistent viruses are acquired and transmitted to the plant during brief probes (lasting less than 1 min), do not require a latent period in the vector and are retained in the vector for only short periods of time (aphids remain viruliferous for only a few hours). Persistent viruses are acquired during phloem punctures for feeding (over periods of several hours or even days); they have a latent period and are retained for long periods in the vector (aphids often remain viruliferous for life). Further information about the transmission of plant viruses by insects is available from a recent review (Fereres and Raccah, 2015).

Non-persistent viruses include the CMV, belonging to genus *Cucumovirus*, family *Bromoviridae.* This virus has a worldwide distribution, and has been observed to infect more than 1200 species from more than 100 plant families (Jacquemond, 2012). This virus may have the widest host range of any known plant virus. It can be transmitted by more than 80 aphid species. CMV causes typical mosaic symptoms on melon leaves, plant stunting, mottle or mosaic on fruits, and yield losses.

Several potyviruses (family *Potyviridae*) attacking melon crops are also non-persistent (Lecoq and Desbiez, 2012). They are transmitted by 20 to 40 aphid species. *Watermelon mosaic virus* (WMV) is observed worldwide. It can infect more than 170 plant species. On melon leaves, it induces mosaic, vein banding and deformation, such as blisters, filiformis and size reduction. On fruits, it induces severe discoloration, with slight deformation in some cases. *Zucchini yellow mosaic virus* (ZYMV) is also distributed worldwide, but has a smaller host range than CMV or WMV (only 11 families). It induces vein clearing, yellowing, with blisters and enations on leaves and severe stunting. On fruits, ZYMV induces mosaic or necrotic cracks, marbling and hardening of the flesh. Moreover isolates belonging to the pathotype F induce wilting in melons carrying the *Fn* gene (Risser et al., 1981) instead of mosaic in melons carrying the *Fn*^+^ allele. The *Fn* gene (for *Flaccida necrosis)* is present in numerous melon accessions. *Papaya ringspot virus* (PRSV) mostly infects tropical and Mediterranean cucurbit crops. Its host range is restricted to cucurbits and a few other plant species, such as papaya. On melon, it causes severe mosaic, blistering, and malformations on leaves. Fruits may also display various degrees of discoloration and deformation.

Persistent viruses include the *Cucurbit aphid-borne yellows virus* (CABYV) a member of genus *Polerovirus*, family *Luteoviridae* (Lecoq and Desbiez, 2012). This virus infects many cucurbits, beet, lettuce and many weed species. It is transmitted by a small number of aphid species (*M. persicae* and *Macrosiphum euphorbiae* are additional vectors). It induces yellowing of the older leaves, but complete discoloration of the plant is observed with some melon cultivars. Its effect on yield is less marked than other viruses infecting melon, particularly as it has no effect on fruit quality, instead inducing flower abortions and reducing the number of fruits per plant.

### Keep in Mind Some Features When Considering the Double Phenotype

The double-resistance phenotype elicited by *A. gossypii* has been identified in all seven genetic groups in *C. melo* (**Table 1**). It has been identified in wild accessions from Africa, PI 482398 and HSD2455, both of which have some cultivated characteristics, but it has not yet been identified in wild accessions from Asia. Conversely, most of the accessions and landraces displaying the double phenotype are native to Asia. The double phenotype has been observed in all botanical groups of this species in Asia. We use the term ‘*Vat* melon line’ here to refer to any accession or line displaying this double phenotype in a study.

The aphid clones used in bioassays characterizing the double phenotype are only rarely mentioned. Molecular markers for their identification are available (Vanlerberghe-Masutti et al., 1999) but are still only rarely used to characterize the *A. gossypii* clones used in bioassays. A DNA-reference clone set, at least from clones belonging to the three cucurbit clusters, should be established by the scientific community and made available. Aphids assigned to clusters I and III are the most frequently used (**Table 1**). No data have been published concerning the capacity of clones that are not able to colonize Cucurbits, to elicit resistance to viruses in *Vat* melon.

Resistance to viruses when the melon aphid inoculates the plant with virus has been documented principally for CMV. For example, all the accessions mentioned in **Table 1** displayed the double phenotype when using this virus in the bioassays. In PI 161375 and PI 414723, the resistance to viruses is fully effective against other potyviruses, such as PRSV (formerly known as, WMV1), WMV (formerly known as, WMV2) (Lecoq et al., 1980), and ZYMV (formerly known as, MYSV) (Risser et al., 1981; Kishaba et al., 1992; Soria et al., 2000), when the melon aphid inoculates the plants. Like CMV, these viruses have a non-persistent mode of transmission. While not formally tested, it is likely that this large spectrum of resistance to viruses transmitted in a non-persistent manner is common to all accessions displaying resistance to CMV transmission from *A. gossypii*. For other virus species transmitted in a persistent manner, such as CABYV, no laboratory data have ever been published.

## From *Vat* Gene to *Vat* Cluster

**FIGURE 1:**
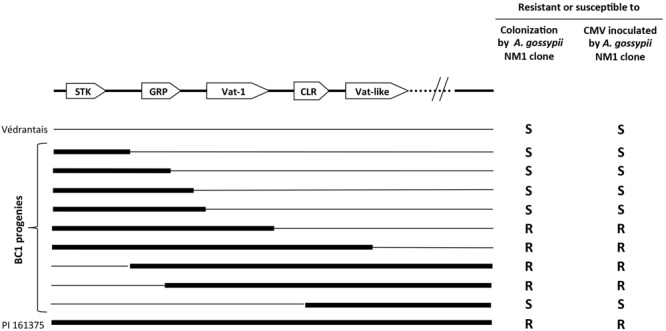
**Genomic map spanning the *Vat* region in 9 Back-Cross 1 plants derived from a cross between PI 161375 and Védrantais displaying segregation for resistance to *Aphis gossypii* and resistance to viruses transmitted by *A. gossypii*.** STK, GRP, and CLR indicate serine-threonine kinase, glycine-rich protein and copia-like retroelement proteins, respectively. Adapted from (Dogimont et al., 2014).

**FIGURE 2:**
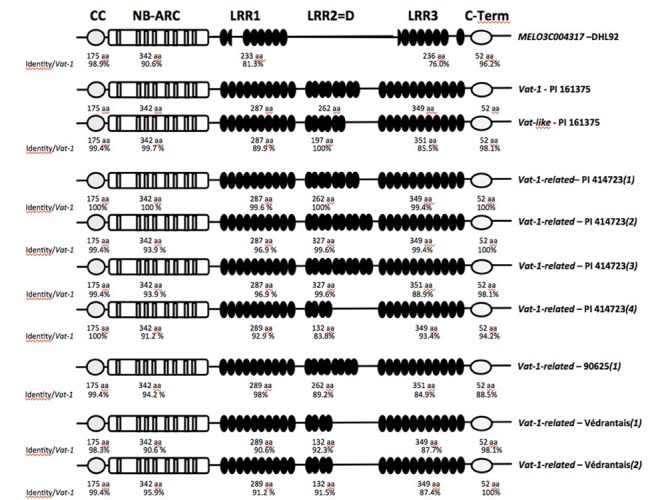
**Schematic diagrams of predicted *Vat* protein domains encoded by the *Vat-1* gene and polymorphisms detected in related sequences.** Adapted from Dogimont et al. (2008a, 2014) and González et al. (2014).

The *Vat* locus was mapped to *C. melo*’s linkage group V using segregating populations from a cross between the susceptible line Védrantais and the resistant accession PI 161375 (Pitrat, 1991; Baudracco-Arnas and Pitrat, 1996). It was localized to a subtelomeric position on a saturated map combining two recombinant inbred populations resulting from crosses between Védrantais and two resistant accessions PI 161375 and PI 414723 (Périn et al., 2002). In early 2000, a map-based strategy was used to isolate the *Vat* gene. This approach involved the use of 6000 plants from a back-cross population derived from Védrantais and PI 161375. Recombination events within the terminal region of linkage group V were screened and recombinant plants were phenotyped for resistance to aphids. A physical map encompassing the *Vat* gene was obtained by screening a melon bacterial artificial chromosome (BAC) library constructed from PI 161375, and the genomic sequence spanning the *Vat* region was annotated. A comparison of molecular data and phenotypic data for resistance to melon aphid and resistance to viruses when the melon aphid inoculated the viruses showed that the *Vat* gene was a single functional locus conferring both types of resistance (Pauquet et al., 2004). Nine of the 14 of the back-cross progeny displaying recombination in the genomic sequence spanning the *Vat* region are presented in **Figure 1**, with their phenotype. The *Vat* gene is 6-kb long, and consists of five exons and four introns. It encodes a predicted 1467-amino acid protein presumed to be located in the cytoplasm (Dogimont et al., 2014). This protein belongs to the coiled-coil (CC)-nucleotide binding site (NBS)-leucine-rich repeat (LRR) family (**Figure 2**). Only three other genes conferring resistance to aphids or other hemipterans, *Mi-1* in tomato and *Bph14* and *Bph26* in rice are known to encode proteins from the NLR family (Rossi et al., 1998; Nombela et al., 2003; Casteel et al., 2006; Du et al., 2009; Tamura et al., 2014). For confirmation of the effect of the *Vat* resistance allele, an 11-kb DNA fragment harboring *Vat*’s coding region, promoter, and 3′-flanking region, was introduced into two susceptible *C. melo* lines by *Agrobacterium*-mediated transformation (Dogimont et al., 2014). Four lines derived from independent transformation events were obtained and all lines displayed high levels of resistance to NM1 melon aphids and complete resistance to viruses when the NM1 aphids inoculate the transgenic plants with CMV, WMV, and ZYMV.

All the bioassays conducted to identify *Vat* in the melon genome to date have used NM1. This clone has been used since the early studies by the French team and has provided the most clear-cut differentiation between susceptible and resistant accessions for both resistance to melon aphid and resistance to the viruses introduced into the plant by melon aphid (Boissot et al., 2016). In a study investigating whether the *Vat* allele of PI 161375 had a specific aphid clone effect or a much broader effect, one of the transgenic lines was tested with a set of *A. gossypii* clones from the cucurbit host-race. The bioassay used assessed the resistance to viruses transmitted by the aphid. In the transgenic line, the resistance to viruses introduced by an aphid was aphid clone-specific. Surprisingly, for some clones used for inoculation purposes, resistance to the virus was expressed in the native line, PI 161375 but not in the transgenic line (**Table 2**). These remarkable differences reveal that at least one other gene is involved in the resistance elicited by some *A. gossypii* clones in PI 161375 (Boissot et al., 2016). In accordance with general rules for the naming of genes, it has been suggested that the gene isolated from PI 161375 (Dogimont et al., 2014) should be renamed *Vat-1*, and the additional gene *Vat-2*. There may be allelic series for both these loci (Boissot et al., 2016).

**Table 2:** Pattern of resistance to *Cucumber Mosaic Virus* (CMV) inoculated by six *A. gossypii* clones on PI 161375, from which *Vat-1* was isolated, TR3, the transgenic line in which it was introduced and Margot a line in which aphid resistance from PI 161375 was introduced by classical breeding.

	C6	GWD	CUC1	C9	GWD2	NM1
PI 161375	S	R	R	R	R	R
TR3	S	S	I	R	R	R
Margot	S	R	R	R	R	R

*R, resistant; S, susceptible; I, intermediate.*

It is not clear from the results presented above whether *Vat-1* and *Vat-2* form a cluster. This point has been investigated indirectly. PI 161375 is a Korean line harboring *Vat-1* and *Vat-2*; it belongs to the Chinensis botanical group (**Table 1**). *Vat-1* was introgressed from this line into a Charentais line (Cantalupensis group). The process of introgression consists in a first crossing between a Charentais line and PI 161375 and after, backcrossing the aphid-resistant progeny with the Charentais line, referred to as the recurrent line. The bioassays, to select plants resistant to aphids from each back-cross progeny, used the aphid clone NM1. Remarkably, the spectrum of resistance to viruses transmitted by aphids in PI 161375 was found to be conserved in the line Margot, which was obtained after seven back-crosses, (**Table 2**) and therefore Margot carries *Vat-2* (Boissot et al., 2016). This suggests that *Vat-1* and *Vat-2* are probably very tightly linked. We will therefore use the name ‘*Vat’* for the region containing *Vat-1* and *Vat-2*.

Several genomics studies have focused on the region containing *Vat*. Genes conferring resistance to various pathogens are located in the vicinity of *Vat*: resistance to *Podosphaera xanthii* (Yuste-Lisbona et al., 2001; Perchepied et al., 2005a), *Cucumber vein yellowing virus* (Ibn Oaf, 2012), the *Fn* gene (Pitrat and Lecoq, 1982) triggering plant necrosis in response to some isolates of *ZYMV* (Risser et al., 1981), and the quantitative trait loci (QTL) *FomV-2* conferring partial resistance to *Fusarium oxysporum* f. sp. *melonis* (Perchepied et al., 2005b). The density of resistance genes in melon is highest in the region containing *Vat* (Garcia-Mas et al., 2012); 28 genes of the NLR family have been identified in a 1-Mb region containing *Vat* (González et al., 2014). Characterization of four *C. melo* accessions displaying resistance to viruses when different *A. gossypii* clones inoculated the plants has identified *Vat-1*-related sequences (protein identity over 80%; **Figures 1** and **2**).

These sequences display polymorphisms within all parts of the gene (**Figure 2**). In the LRR part of *Vat*, two types of polymorphism are observed: single-nucleotide polymorphisms (SNP) and length polymorphisms. The length polymorphisms occur in a specific domain, domain D or LRR2 (**Figure 2**) (Dogimont et al., 2008a, 2014). This domain consists of near-perfect repeats of 65 amino acids. The *Vat-1-*related sequences contain two to five repeats. The repeats are 83.1–89.2% identical (Dogimont et al., 2008a). PI 161375 has a *Vat-1-*related sequence with three repeats known as *Vat*-like. *Vat*-like is located 17 kb from *Vat-1* (**Figure 1**) and is not involved in the resistance elicited by the NM1 clone (Dogimont et al., 2008b). In PI 414723, a line exhibiting resistance to several clones, four *Vat-1-*related sequences have been identified. One of these sequences has only a few SNPs relative to *Vat-1*. Two sequences have five repeats in LRR2, and both are strong candidates for the control of resistance to *P. xanthii* (Dogimont et al., 2008b). Both these *Vat-*related sequences have few SNPs relative to the sequence of *Vat-1.* The fourth *Vat-1-*related sequence has two repeats in LRR2 and more SNPs relative to *Vat-1* than the other *Vat-1-*related sequences. In 90625, a line exhibiting resistance restricted to only one clone, NM1 (Boissot et al., 2016), only one *Vat-1-*related sequence has been identified. This sequence contains four repeats in LRR2 and several SNPs. In Védrantais, a Charentais line resistant to viruses when only one aphid clone, C4, inoculate the plant (Boissot et al., 2016), two *Vat-1-*related sequences with several SNPs relative to the sequence of *Vat-1* and two repeats in LRR2 have been identified. Finally, the cadre of *Vat*-like genes in each accession may not be complete, since no complete genome sequences are available for these accessions. Involvement of these *Vat-1-*related sequences in the aphid resistance has not been demonstrated. Actually, the melon reference genome was built from a line (DHL92) given as susceptible to *A. gossypii* (González et al., 2014), but accurate double-phenotypic data, with a set of characterized clones, are missing for that line. This line has a *Vat-1* homolog, MELOC004317, shorter (1038 aa) than the reference one (1467 aa), in particular the LRR2 part is fully absent (**Figure 2**) (González et al., 2014).

The presence of large numbers of NLR genes in close proximity to *Vat*, including Vat-like genes, makes assembly difficult and even unsatisfactory when sequences are obtained for small fragments. Sequencing studies of a set of accessions are required with longer fragments for an accurate assembly of the area. Comprehensive cross-comparison between molecular and phenotypic data is therefore required to obtain a full understanding of the genetic control of resistance to *A. gossypii* aphids and the viruses they transmit. The use of transgenic lines, even if difficult to obtain (Chovelon et al., 2011), will clearly help us to decipher the role of each locus in this cluster. As soon as candidates are identified, transformation with these candidates could be used to validate their roles in the resistance spectrum. This approach could provide new opportunities for genomic selection for resistance in melon.

## Molecular Responses in the *Vat* Melon*/A. gossypii* Aphid Interaction

**FIGURE 3:**
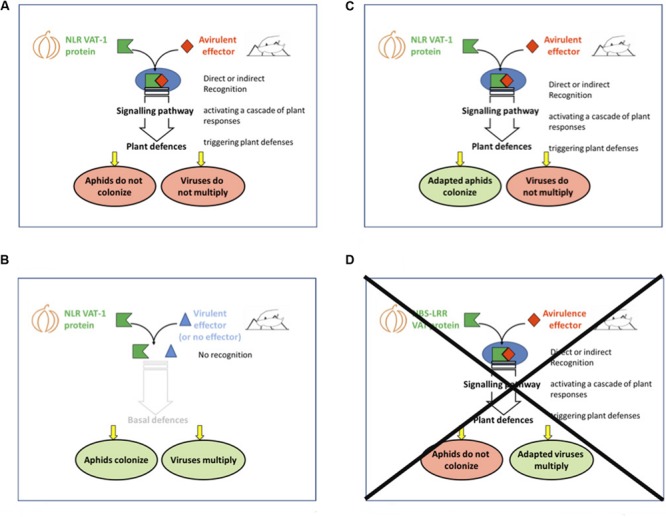
**Models of *A. gossypii*/*Vat*-melon plant interaction based on interaction (direct or not) between the VAT protein and the avirulence effector (Boissot et al., 2016).** The three cases observed were: **(A)** resistance to aphids and viruses, **(B)** susceptibility to aphids and viruses, **(C)** susceptibility to aphids and resistance to viruses. **(D)** A fourth outcome, resistance to aphids and susceptibility to viruses, was not observed.

The *Vat-1* gene belongs to the NLR gene family. According to the general framework developed for this category of resistance genes, its functioning involves separate recognition and response phases (**Figure 3A**). In this case, the recognition phase involves perception of an aphid effector by the plant’s VAT protein, which may be direct or indirect. The vast majority of plant-pathogen effectors have been shown to interact indirectly with a NLR protein of their host. To date, modality of the interaction between aphid effectors and NLR proteins of their host is still unknown. This interaction should occur in the cytoplasm of cells (the predicted location of the VAT protein), and the effector molecule must therefore be delivered to the plant cell. Aphid mouthparts enclose two flexible stylets that the insect drives into the plant tissue to puncture the phloem, so that they can feed on plant sap. On their way to the phloem, the aphid stylets bend around the cells, and the salivary channel ejects a salivary gel that forms a sheath around the stylets. Along the way to their destination, the aphid stylets briefly puncture cells, into which the salivary channel ejects a watery saliva (Tjallingii, 2006). The effector is probably injected into the cells during these puncture events. No aphid effector capable of interacting with an NLR protein has yet been identified. A transcriptomic approach has been used to identify candidates involved in the virulence of *A. gossypii* on *Vat* melon (Dutartre-Fricaux et al., 2014). Genetically similar virulent and avirulent clones were used, and a head-reference transcriptome of more than 33000 contigs was generated by *de novo* assembly. This reference transcriptome has been used to search for candidate effector genes based on their differential expression and/or presenting sequence polymorphisms between virulent and avirulent aphid clones.

At the molecular plant level, the response phase is thought to involve the activation of a signaling cascade, leading to the rapid accumulation of reactive oxygen species (ROS) and defense hormones. In a *Vat* melon line, the levels of miRNAs involved in the post-transcriptional regulation of gene expression change rapidly after puncture by *A. gossypii* (Sattar et al., 2012b). Within 12 h of the infestation by melon aphids, 23 families of miRNAs display modulations. Their potential targets suggest a physiological function in disease and stress responses (5), phytohormone perception and signaling (11), miRNA biogenesis (2), and plant growth and development (Sattar et al., 2016). Ethylene, jasmonic acid and auxin have been identified as potential defense hormones in *Vat* plants infested with *A. gossypii* (Anstead et al., 2010; Sattar et al., 2016). In two *Vat* melon lines, peroxidase activity was found to increase within 10 min of aphid puncture (Sarria Villada et al., 2009). Callose, a polysaccharide usually laid down at plasmodesmata, is deposited within 20 min of aphid infestation, and lignin, a macromolecule derived from phenyl propanoids essential for cell wall thickening, is deposited 4.5 h after aphid infestation. The plasma membrane is damaged and the cells collapse. Callose and lignin are deposited in the wall of cells adjacent to the stylet sheath. These reactions do not occur in non-*Vat* plants. These data clearly illustrate the massive transcriptional reprogramming induced by *A. gossypii* infestation in *Vat*-resistant melon plants, triggering a wide range of plant defense responses (Dogimont and Boissot, 2016; Sattar et al., 2016).

At the molecular aphid level, changes in gene expression were investigated in *A. gossypii* feeding on *Vat* and non-*Vat* plants. There is an unexpectedly high abundance of 27 nt-long sRNA sequences in aphids feeding on *Vat* plants (Sattar et al., 2012a). These sRNAs belong to the Piwi-interacting RNA (piRNA) family. This class of sRNAs is absent from plants. Their biogenesis in animals is still poorly understood. They have been shown to be involved in the silencing of transposable elements exclusively in animal gonads (Vodovar and Saleh, 2012), facilitating short-term adaptation. Their role in *A. gossypii* remains unknown, but may relate to the lifting of maternal effects. Such effects are observed, for example, in aphids collected from cotton, which have low rates of reproduction in the first generation after transfer onto eggplant, but higher rates in subsequent generations (Satar et al., 2013). Eighty-one conserved microRNAs (miRNAs), 12 aphid-specific miRNAs, and nine novel candidate miRNAs have also been identified (Sattar et al., 2012a). These candidate miRNAs have been shown to be differentially regulated between aphids feeding on *Vat* and non-*Vat* plants and may affect their reproductive rates as described below.

## Effect of *Vat* Plant Responses on *A. gossypii* Behavior and Biology and the Virus they Transmit

Aphid feeding is disrupted on *Vat* plants. Electrical-Penetration graph (EPG), in which the pathway of aphid stylets from epidermis to phloem can be followed, have been conducted on several *Vat* melon lines, with different genetic backgrounds and on melon aphid clones originating from different geographic areas (Kennedy et al., 1978; Chen et al., 1997; Klingler et al., 1998; Boissot et al., 2000; Garzo et al., 2002). The journey of the stylets through the mesophyll to the phloem takes from 90 to 140 min in non-*Vat* plants (Kennedy et al., 1978; Chen et al., 1997; Klingler et al., 1998; Boissot et al., 2000; Garzo et al., 2002), but is disrupted in *Vat* plants. The observed cellular response seems to occur after the aphid stylets have punctured plant cells rather than during the intercellular penetration of plant tissues by the stylets (Sarria Villada et al., 2009), consistent with the hypothesis that recognition occurs after the delivery of the effector to the cell. Cytological studies have shown that there are more stylet sheaths in *Vat* plants than in non-*Vat* plants (Kennedy et al., 1978), suggesting that early mesophyll cell puncture by *A. gossypii* may be more frequent in *Vat* plants. The stylets take longer to reach the phloem in *Vat* plants than in non-*Vat* plants and are less likely to reach their final destination in *Vat* plants than in non-*Vat* plants. Prior exposure of *Vat* plants to *A. gossypii* feeding does not modify the expression of this resistance (Chen et al., 1997).

Findings on *Vat* melon suggest that the plant responses elicited by short cell punctures either hinder the passage of the stylets between cells due to the deposition of callose and/or lignin in the cell walls, or deter the aphid from progressing further into the tissues, through an oxidative burst detected by aphid after brief periods of ingestion following the release of saliva into the cell. Moreover, melon aphids reaching the phloem of *Vat* plants do not remain there to feed (less than 10 min, if at all; Kennedy et al., 1978; Chen et al., 1997; Klingler et al., 1998; Boissot et al., 2000; Garzo et al., 2002). This suggests that feeding may be difficult in these plants, possibly due to phloem clogging, although this plant reaction has not yet been described in *Vat* melon. Few quantitative differences in phloem sap have been identified between *Vat* and non-*Vat* plants that might explain the deterrence of *A. gossypii* (Chen et al., 1997). None of the studies, EPG or histological studies, investigating these aspects took into account the dual phloem structure of cucurbits and the nature of the phloem in which *A. gossypii* is able to establish feeding.

Aphids escape from *Vat* plants. In free-choice tests, winged aphids are less numerous on *Vat* plants 24 h after being offered a choice of plants (Kennedy and Kishaba, 1977), and wingless aphids are less numerous on *Vat* leaf disks from 30 min after being offered a choice of leaf disks (Garzo et al., 2002). Without choice, i.e., only one plant accession is available, wingless aphids walk away from the plant in the 2–3 days after their deposition. This behavior has been observed on several *Vat* melon lines, with aphid clones originating from different geographic areas that probably displayed marked genetic differences (Pitrat and Lecoq, 1982; Garzo et al., 2001; Boissot et al., 2010; Thomas et al., 2012b). Based on the timing of these events, we can conclude that early plant responses, occurring rapidly after cell puncture by the aphid, have an immediate effect on aphid behavior.

Aphids poorly reproduce on *Vat* plants. When wingless aphids are encaged on *Vat* plants, they display low rates of reproduction, mostly due to a longer pre-reproductive period and a smaller number of progeny (Klingler et al., 1998; Garzo et al., 2002; Boissot et al., 2010). This lower reproductive rate may be directly due to poor, disrupted feeding on the contents of phloem (Kennedy et al., 1978; Chen et al., 1997; Klingler et al., 1998; Boissot et al., 2000; Garzo et al., 2002). This hypothesis is supported indirectly by the observation that aphids produce far less honeydew when feeding on *Vat* plants than on non-*Vat* plants (Klingler et al., 1998). The resistance factor reducing the reproductive rate is not transmitted through grafting (Kennedy and Kishaba, 1977), consistent with the notion that feeding is somehow difficult, rather than with the phloem being toxic. The rate of aphid reproduction is quantitatively affected by *Vat* and QTLs (Kishaba et al., 1976; Boissot et al., 2010; Thomas et al., 2012b).

*Vat* plants display particularly high levels of resistance to viruses when aphids inoculated unrelated viruses transmitted in the non-persistent mode: 100% of the non-*Vat* plants displayed symptoms, whereas only 0 to a few per cent of *Vat* plants had symptoms, with this small number of plants displaying full symptoms (Lecoq et al., 1979; Kishaba et al., 1992; Boissot et al., 2016). When *Vat* resistance was first discovered, it was thought to block virus transmission, and Pitrat and Lecoq therefore named the gene responsible *Vat*, for ‘virus aphid transmission’ (Pitrat and Lecoq, 1982). Nevertheless, *A. gossypii* was subsequently shown to acquire the virus from *Vat* plants and to transmit it to non-*Vat* plants (Romanow et al., 1986), calling this hypothesis into question. According to the resistance to transmission hypothesis, a plant factor blocks the virus in the stylet (a molecule or a particular pH). The aphid must, therefore, first ingest material from the plant, before it egests saliva into the cells and delivers the viruses. All EPG studies have shown that, after puncturing cells, the aphid first salivates and then ingests the contents of the cell (Martin et al., 1997). Further studies have failed to demonstrate resistance to transmission due to retention of the virus in the stylets.

*Vat* resistance to viruses has to be considered in the general framework described for NLR resistance (Boualem et al., 2016). The VAT protein of non-*Vat* plants (i.e., carrying a ‘susceptible’ *Vat* allele) cannot recognize (directly or otherwise) the aphid effectors and viruses delivered to the cell by the aphid. This lack of recognition leads to systemic viral infection. In *Vat* plants, the resistant isoform of VAT recognizes an effector molecule from the aphid. This recognition induces resistance mechanisms limiting the replication and movement of the virus. The micro-oxidative burst triggered by aphid puncture in *Vat* plants (Sarria Villada et al., 2009) is thought to block the viruses in the inoculated cell or in neighboring cells. Callose deposit at plasmodesmata may help to contain virus particles in the inoculated or neighboring cells. The response is local: when a *Vat* plant is first inoculated with CMV by *A. gossypii*, CMV superinoculation with *M. persicae* on the same leaf leads to systemic infection (Mistral and Boissot, 2016). In the absence of the aphid effector, the recognition phase does not occur when the *Vat* plant is infected with viruses. In this case, the viruses replicate and move around the plant, establishing a systemic virus infection.

Virus aphid transmission (the initial name from which the Vat acronym is derived) does not provide an accurate picture of the action of the *Vat* gene product according to recent data. It would, therefore, be more appropriate to consider *Vat* to stand for ‘virus aphid triggered.’

In *Vat* plants, no resistance to viruses transmitted in the persistent mode has ever been reported. As CABYV is restricted to phloem cells, the aphid must reach the phloem cells and feed for long enough to acquire virus particles. Mechanical inoculation is not possible for this virus, suggesting that effective inoculation is dependent on the delivery of the virus directly into the phloem. As *A. gossypii* rarely reaches the phloem of *Vat* plants (Kennedy et al., 1978; Chen et al., 1997; Klingler et al., 1998; Boissot et al., 2000; Garzo et al., 2002), CABYV acquisition and inoculation should be disrupted in *Vat* plants, but this point needs to be investigated.

## Ability of *A. gossypii* and the Viruses it Transmits to Adapt to *Vat*

The LRR domain of the *Vat-1* gene is subject to diversifying selection (Dogimont et al., 2014). This selection responds to the diversifying selection acting on the avirulence gene, as frequently reported for other avirulence genes in plant pathogens (Rouxel and Balescent, 2010). This model describes the general framework for the molecular arms race between plants and pathogens. An aphid clone adapted to a given *Vat* allele would either not deliver an ‘avirulent’ effector to the plant (deletion) or would deliver a ‘virulent’ effector that is not recognized by the VAT protein. In both cases, the expected phenotype would be colonization of *Vat* plant by the clone, and susceptibility to viruses introduced into the plant by that aphid clone (**Figure 3B**).

This model has been challenged by testing transgenic lines containing the *Vat-1* gene, for resistance to both *A. gossypii* and CMV introduced into the plant by six *A. gossypii* clones (C6, C9, CUC1, GWD, GWD2, and NM1) (Boissot et al., 2016). The phenotypes for five of the six clones were consistent with the general model (**Figures 3A,B**): the clones were either unable to fully colonize the *Vat-1* transgenic line and triggered resistance to CMV (NM1, C9, **Figure 3A**), or they fully colonized the *Vat-1* transgenic line and did not trigger resistance to CMV (C6, CUC1, GWD, **Figure 3B**). These five clones belonged to the three clusters corresponding to the cucurbit host-race (**Table 1**). The phenotypes observed with one clone (GWD2) were not consistent with the general model, with the clone triggering resistance to viruses but nevertheless being able to colonize the transgenic plants (**Figure 3C**).

Unlinking of resistance to viruses triggered by the aphid and resistance to aphids was confirmed by testing eleven *Vat* lines identified from the natural range of diversity in melon with nine clones, the six previously described and CUC6, CUC3, and C4 (Boissot et al., 2016). Only 52 of the 117 interactions characterized, considering results on transgenic lines and natural *Vat* lines, were consistent with the general model (**Figures 3A,B**). It has been hypothesized that the decoupling of the resistances to aphids and viruses (**Figure 3C**) results from aphid adaptation, making it possible for the aphid to colonize the plant even if plant defenses are elicited. The *Vat* phenotype proved to be a highly powerful tool for investigating a phenomenon never before studied for plant/pathogen interactions. This new model for adaptation to NLR resistance is revealed by the double phenotype, which can be used to follow the resistance process at two levels: recognition, and the efficacy of the plant defenses triggered.

We speculate that individuals of some clones adapted to *Vat* defenses they trigger (e.g., GWD2). If these aphids infest a *Vat* plant successfully, they must accept the *Vat* plant and reproduce at a high rate on it. EPG has revealed that *Vat* affects the exploratory behavior of the aphid on the plant, but this effect is quantitative, with some aphids reaching the phloem of *Vat* plants. Individuals of a clone adapted to *Vat* defenses probably reach the phloem more often than those of a non-adapted clone. A ‘classical avenue’ of research will involve comparison of the transcriptomes of adapted and non-adapted clones puncturing *Vat* plants, to track the aphid genes involved in this adaptation. We propose to explore a new avenue of research: does the dual phloem structure of cucurbits, and of melon in particular, play a role in this adaptation?

It is possible *a priori* that viruses transmitted in the non-persistent mode can overcome *Vat*-mediated resistance. In this scenario, viral variants may multiply and leave the punctured cells before the defense mechanisms are fully effective, resulting in the development of systemic infections. The expected phenotype would be ‘susceptibility to viruses introduced by an aphid clone that is incapable of colonizing *Vat* plants’ (**Figure 3D**). To date, this double phenotype has never been observed in a transgenic line carrying the *Vat-1* gene, suggesting that viral adaptation may not occur (Boissot et al., 2016). Consistent with these findings, experimental evolution experiments with viruses on *Vat*-plants have been unsuccessful. Sequential virus transmissions from infected *Vat* melon plants to healthy *Vat* melon plants were established with two aphid clones and three viruses, CMV, ZYMV, and WMV. None of these viruses evolved in response to the resistance triggered by these two clones, even when four sequential virus transmissions could be done (Boissot et al., 2016). These results strongly suggest that viruses transmitted in the non-persistent mode do not readily adapt to the *Vat* resistance triggered by *A. gossypii*.

## Effect of *Vat* on *A. gossypii* at the Population Level and its Durability

**FIGURE 4:**
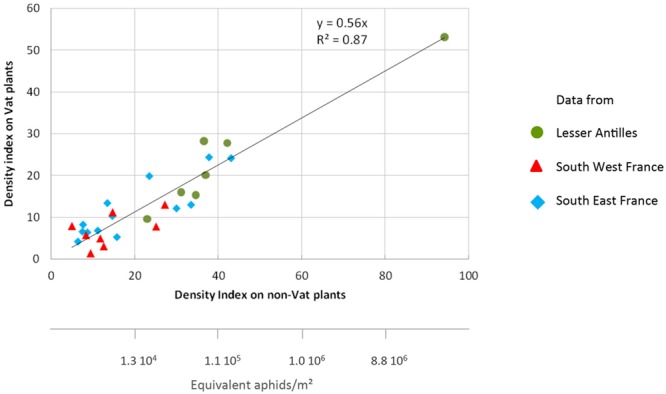
**Aphid densities on *Vat* and non-*Vat* plants grown in 28 fields in three melon production areas from 2008 to 2014.** The data shown are from (Schoeny et al., 2014; Thomas et al., 2016).

Aphid density is lower on *Vat* plants than on non-*Vat* plants (Thomas et al., 2016). With the aim of quantifying the effect of *Vat* at crop level, we compiled bibliographic data for a density index for *Vat* and non-*Vat* plants grown in field experiments conducted at the same location (Schoeny et al., 2014; Thomas et al., 2016). The aphid density index was 44% lower on *Vat* plants (**Figure 4**). This index is related to aphid density per m^2^ over the entire cropping period, by an exponential relationship (*y* = 1463e^0.1088x^ with *r*^2^ = 0.72, *n* = 304). Therefore, for non-*Vat* plant indexes of 90, 50, and 30, aphid density is reduced by factors of 50, 11, and 4, respectively, on *Vat* plants.

**FIGURE 5:**
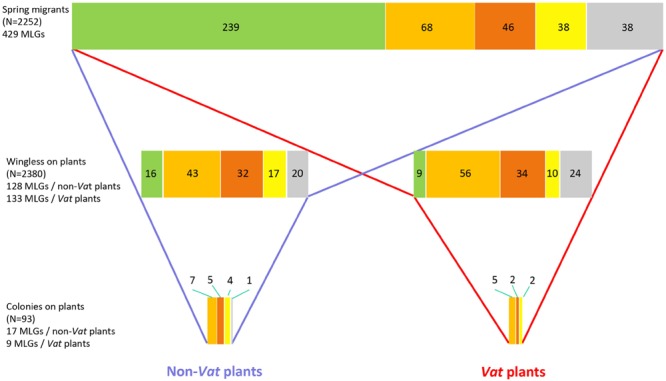
**Reduction of the clonal richness of *A. gossypii* populations during the infestation of melon crops in France.** Clonal richness at each step of the infestation is represented by the length of the stripe. Colors within the stripe represent different genetic clusters of *A. gossypii* populations, with the size of each rectangle proportional to the number (indicated within) of MLGs assigned to the genetic cluster. The data are available from the Dryad Digital Repository: http://dx.doi.org/10.5061/dryad.gf54q 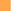
Cucurbit I, 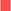
Cucurbit II, 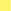
Cucurbit III, 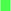
other clusters, 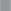
Not assigned at 75%.

There are four key phases in the dynamics of crop infestation by aphids: visiting by winged aphids, infestation with the wingless nymphs they generate, development into colonies, and production of winged individuals for dispersal. Melon crops are visited by spring migrant aphids of numerous species. The proportion of *A. gossypii* among the visiting aphids and the genetic composition of the *A. gossypii* spring migrant population depend on geographic area (Thomas et al., 2016). Only some of the *A. gossypii* spring migrants generate progeny (**Figure 5**), mostly specializing on cucurbits (i.e., belonging to the Cucurbits I, II, and III genetic clusters). This selection leads to a significant decrease in clonal richness between the spring migrant and wingless populations on melon plants (Thomas et al., 2012a), and this decrease continues during subsequent steps, reflecting differences in fitness or competition between clones on melons (Thomas et al., 2016) (**Figure 5**).

The effect of *Vat* on the first step of infestation has never been reported at field level. However, *Vat* plants were found to be less attractive for winged aphids than non-*Vat* plants in greenhouse experiments based on artificial infestation (Kennedy and Kishaba, 1977). The effect of *Vat* on subsequent phases of infestation has been characterized in open-field melon crops under natural infestation conditions, but only in French melon production areas and in the Lesser Antilles (Thomas et al., 2016). The wingless populations on *Vat* plants have a genetic composition different from that of the populations on non-*Vat* plants, as clonal richness and clonal diversity decreased during infestation. Aphids from group III of the cucurbit host-race are eliminated in favor of aphids from group I. In French production areas, the third step, colony development, is erratic on melon crops, and, in the presence of *Vat*, this step is very rarely observed, generally in only a few aphid genotypes from group I or II. The fourth phase, the production of winged individuals for dispersal, is density dependent (Dixon, 1985), and therefore very rare on *Vat* plants. The populations of *A. gossypii* occurring in French melon crops contain a large proportion of aphids from genetic group III, and the decrease in density and diversity on *Vat* plants probably reflects selection rather than competition. Consistent with this hypothesis, a laboratory study showed that *Vat*-mediated resistance affected the population growth of 90% of group III clones, but only 40% of those from group I or II (Lombaert et al., 2009). Only group I aphids are present in the Lesser Antilles, where the third and fourth steps are regularly reached, even on *Vat* plants. The effect of *Vat* on aphid clonal richness is illustrated in **Figure 5.** Only four clones, CUC1, CUC6, GWD, and C6, have regularly been identified in colonies on *Vat* plants in field experiments (Thomas et al., 2016) and studied in laboratory experiments (Boissot et al., 2016). C6 does not trigger resistance to CMV and probably has a virulent effector not recognized by *Vat*, whereas CUC1, CUC6, and GWD trigger resistance to CMV and are adapted to plant response.

An ecological genetics analyze of melon-aphid dynamics has been applied in three different agricultural systems over the last decade, to predict the durability of *Vat* resistance to *A. gossypii* (Thomas et al., 2016). It appears that *A. gossypii* is evolving at a regional level in response to the deployment of *Vat* melon crops. For example, two different bottlenecks affect the dynamics of adapted clones in melon-producing areas, due to (i) the low levels of dispersal morph production on *Vat* melon and (ii) the winter extinction of clones. The low levels of dispersal morph production result from the containment of populations at levels of crowding below that required to induce the production of winged morphs. Winter extinction occurs due to the absence of other cucurbit crops to serve as hosts between two melon crop cycles, limiting the maintenance of *Vat*-adapted clones. In melon-producing areas without bottlenecks (such as the Lesser Antilles), resistance is predicted to be not durable. In areas in which both types of bottlenecks occur (such as South-West France), resistance is predicted to be durable. In South-East France, only one of the two bottlenecks occurs, and cucurbits are cultivated almost year-round. Moreover, in South-East France *Vat* melons have been cultivated at a large scale since 2000, and *Vat* resistance is now jeopardized by the emergence of adapted clones. These findings suggest that, for a cosmopolitan pest, such as *A*. *gossypii*, decisions concerning resistance deployment should take into account the genetic structure of the pest population at regional scale, the availability of winter host plants for adapted biotypes between crop cycles (Thomas et al., 2016) and the allele composition of the *Vat* cluster.

The manipulation of agricultural systems to increase the durability of *Vat* resistance through winter extinction does not appear to be feasible. However, it may be possible to increase durability by preventing the production of dispersal morphs from adapted clones. Different ways of achieving this aim have been investigated. The use of strips of flowering plants sown close to *Vat* melon crops to attract natural enemies has been investigated (Schoeny et al., 2014), but the efficacy of this approach will need to be confirmed over several years. Alternatively, QTLs decreasing the production of dispersal morphs on *Vat* plants could be sought. The identification of such QTLs is probably feasible in melon accessions displaying resistance to aphids *sensu stricto* (no elicitation of resistance to virus by *A. gossypii*). This phenotype, like classical phenotypes of resistance to aphids described in other crops, has already been observed in the natural range of melon diversity (Boissot et al., 2016). QTLs controlling this phenotype could be combined with *Vat* resistance in a melon breeding program.

## Effect of *Vat* on Viruses at the Population Level and Durability of Resistance to Viruses

The effect of *Vat* on virus epidemics is poorly documented. Field experiments were conducted in France in the late 1970s to compare the development of CMV in *Vat* accession PI 161375 and a non-*Vat* melon cultivar (Lecoq and Pitrat, 1983). CMV progression curves had the same general “S” shape, with a steep slope, but disease onset was always earlier in the susceptible plots, with symptoms observed 12–24 days later in *Vat* plots than in non-*Vat* plots. This evaluation continued into the 1980s, with the resistant cultivar Virgos (Lecoq and Pitrat, 1989). In accordance with previous results, resistance delayed the CMV epidemic development and greatly decreased the rate of disease increase. It should be borne in mind that PI 161375 and Virgos carry composite resistance to CMV: *Vat* and the oligogenic and recessive resistance to ‘common’ CMV strains (Guiu-Aragones et al., 2014). It is, therefore, difficult to determine the actual contribution of *Vat* to the control of CMV epidemics. These experiments were more informative for WMV, because ‘resistance to common CMV strains’ is not effective against WMV. In *Vat* melon plots, WMV epidemics were delayed slightly (by about 5 days), with no significant reduction of the rate of disease increase (Lecoq and Pitrat, 1989).

Recent studies in South-East France compared virus epidemic development in melon lines differing only by the presence/absence of *Vat* (Schoeny et al., 2014; Boissot et al., 2015). In most field trials, *Vat* had a significant effect on CMV epidemics, mostly by reducing the rate of disease increase. It had no effect on WMV epidemics, probably because *A. gossypii* is not the principal aphid vector of this virus.

The partial effect of *Vat* on CMV epidemics is consistent with *Vat* resistance being elicited by only a proportion of the viruliferous aphids visiting melon crops. Indeed, more than 80 aphid species are able to transmit CMV, therefore viruliferous aphids belonging to these species trigger epidemics when they visit *Vat* and non-*Vat* melon crops. Moreover within *A. gossypii*, it remains unclear whether *A. gossypii* not belonging to the Cucurbit host-race can elicit resistance to CMV resistance. Nevertheless, the partial effect of *Vat* on CMV epidemics remains significant, probably because *A. gossypii* is one of the most efficient vectors of CMV based on laboratory experiments.

Finally, the use of *Vat* to control the spread of non-persistent viruses in melon crops is dependent on the importance of viruliferous *A. gossypii* relative to other vector species in the spread of the virus in the crop. The effect of *Vat* is not sufficient for the full control of virus epidemics in crops, but the broad spectrum of this effect and the inability of viruses to adapt to it (Boissot et al., 2016) have made this type of resistance much of a ‘holy grail’ for plant breeders. In the postgenomic era, it may be possible to edit this resistance gene to make it possible for any aphid species to trigger resistance or for resistance to occur without the need for aphid triggering.

Concerning persistent viruses, *Vat* steadily and significantly decreases CABYV epidemics, mostly by delaying them (Schoeny et al., 2014; Boissot et al., 2015). *A. gossypii* is the principal vector of CABYV so, even though this aspect has not yet been investigated in the laboratory, it appears likely that *Vat* affects CABYV transmission by decreasing both acquisition and inoculation rates. The effect of *Vat* on CABYV population genetic diversity has not yet been documented.

*Vat* resistance has only a partial effect on virus epidemics in melon and is not used in that aim by growers. As a matter of fact resistance to viruses *sensu stricto* needs to be integrated in cultivars to control virus epidemics in crops. This type of resistance to viruses has been identified in *C. melo* species, but has generally been little used in plant breeding programs (Pitrat, 2016). If deployed at a large scale in melon crops, such resistance would exert a selection pressure on viruses, placing the durability of the resistance at risk. The utility of combining *Vat* with resistance to viruses *sensu stricto* has been investigated for CMV and CABYV (Boissot et al., 2015). The epidemic data obtained for *Vat* and non-*Vat* melon crops in South-East France have been integrated into a mathematical model of the evolutionary and epidemiological processes shaping the dynamics of a virus population in a landscape composed of a seasonal cultivated compartment and a reservoir compartment containing virus throughout the year (Fabre et al., 2015). Various agro-ecological systems were considered, mimicking the situation of melon crops in South-East France. The deployment of resistance to viruses *sensu stricto* combined with *Vat* would probably be beneficial for CABYV control, but the potential benefit remains uncertain (although certainly not negative) for the long-term control of CMV. Another modeling study has suggested that the maintenance of low-population aphid populations could prevent the emergence of highly virulent CMV+N-satRNA isolates (Betancourt et al., 2016).

## Conclusion

Finally, since the description of the ‘aphid side’ of the pleiotropic phenotype of *Vat* in the late 1960s, each decade has contributed to improvements in our knowledge and use of this amazing gene. The ‘virus side’ of the pleiotropic phenotype was elucidated in the late 1970s, with the breeding and deployment of the first *Vat* cultivars in the 1980s, and mapping in the 1990s. The assignment of this gene to the NLR gene family in the first decade of the 21st century provided clues to its mode of action, which is now at least partially understood. A succession of new technologies over this period provided new insight into the pleiotropic phenotype of *Vat*. Our knowledge of the genetic diversity of *A. gossypii* has also been refined over time. *A. gossypii* genetic diversity presents a major challenge to *Vat* resistance in the field, but also provides us with opportunities to extend our knowledge of the mechanisms underlying *Vat* resistance.

The *A. gossypii*/melon interaction can be investigated within the broader *A. gossypii/*cucurbit interaction, for at least two points. First, the double phenotype conferred by *Vat* makes it possible to investigate this interaction over the subgroup of *A. gossypii* constituting the Cucurbit host-race. How diverse are the *A. gossypii* strains able to elicit resistance to viruses? The VAT protein probably interacts with ligands present in the *A. gossypii* species or in the *A. gossypii* group. Once the avirulence factor interacting with the VAT protein has been identified, it will be possible to perform genetic diversity studies on that factor. Second, the particular structure of the phloem in cucurbits may play a key role in the specialization of *A. gossypii*, an insect feeding on plant sap, on cucurbits and in adaptation to *Vat* resistance, which decreases the access of *A. gossypii* to the phloem.

The double phenotype can also be used as a tool for ‘reading’ the recognition phase independently of the response phase, whether this response is considered in terms of the response of the plant, or that of the aphid. Promising preliminary results have been obtained with this approach, and the double phenotype could be more extensively used for such studies. The observation that some *A. gossypii* clones trigger resistance responses in *Vat* plants and are adapted to this response provides new insight into the capacity of pests and pathogens to adapt to NLR-mediated resistance in plants. The general framework for resistance mediated by such genes is that, within a pest/pathogen species, a clone/isolate is considered to have adapted if it does not trigger NLR-mediated resistance (i.e., there is no recognition phase). The models proposed for *Vat*/*A. gossypii* interaction suggest that aphid clones are adapted to *Vat* plants either because their avirulence factors do not trigger resistance or because they can colonize the plants even if plant defenses are triggered. Does this second mechanism exist in other NLR plant resistance/pest or pathogen interactions? If so, it would complicate the identification of avirulence factors, because adapted and non-adapted pests/pathogens could have identical avirulence effectors interacting (directly or indirectly) with the protein encoded by the resistance gene. It would also call into question the validity of durability modeling approaches based exclusively on gene-for-gene interaction.

The second way in which *A. gossypii* clones can adapt, such that the *A. gossypii* clones can colonize *Vat* plants whilst eliciting resistance to viruses, appears to be the most common mechanism in *A. gossypii* populations developing colonies on *Vat* crops, raising questions about the evolutionary advantages of such a mode of adaptation. This type of adaptation increases the chances of *A. gossypii* being able to colonize a melon crop free of viruses (because the viruses transmitted by this aphid are blocked in *Vat* plants), probably leading to the production of a larger number of progeny. Further studies are required to assess the advantages of these two types of adaptation.

Many different studies of *Vat* resistance have been carried out, revealing the considerable utility of this gene for addressing research questions and the limitations of the use of resistance genes in agriculture. One key question remains: is this gene really unique among the genes conferring resistance to aphids? No other aphid resistance gene has been reported to confer resistance to viruses transmitted in the non-persistent mode. However, it is not clear how many of the many known aphid resistance genes have been tested for effects on viruses.

## Author Contributions

NB conceived the review and built the figures and tables. AS contributed to the introduction’, the presentation of viruses transmitted by *A. gossypii* to melon crops, to molecular features to the effect of *Vat* at the whole-plant level, *Vat* effect on viruses populations and conclusion. She revised the entire manuscript. FV-M contributed to the presentation of *A. gossypii* and the part on *Vat* effect on *A. gossypii* populations. She revised the entire manuscript.

## Conflict of Interest Statement

The authors declare that the research was conducted in the absence of any commercial or financial relationships that could be construed as a potential conflict of interest.

The reviewer IK and handling Editor declared their shared affiliation, and the handling Editor states that the process nevertheless met the standards of a fair and objective review.

## Footnotes

1 http://faostat.fao.org/

2 http://www.geves.fr
